# Prevalence of depression and associated factors among HIV/AIDS Patients on HAART in Okugu Refugee Camp, Gambella, Ethiopia

**DOI:** 10.3389/fpsyt.2025.1550488

**Published:** 2025-05-23

**Authors:** Adane Asefa, Belay Hirpesa, Zufan Asaye, Nigusie Shifera, Wegayehu Enbeyle Sheferaw, Gebremeskel Mesafint

**Affiliations:** ^1^ Department of Public Health, College of Medicine and Health Science, Mizan-Tepi University, Mizan-Aman, Ethiopia; ^2^ Department of Statistics, College of Natural and Computational Science, Mizan-Tepi University, Tepi, Ethiopia; ^3^ Faculty of Health Science, Health Research Institute, University of Canberra, Bruce, ACT, Australia; ^4^ Department of Psychiatry, College of Medicine and Health Science, Mizan-Tepi University, Mizan-Aman, Ethiopia

**Keywords:** depression, mental health, refugees, refugee camp, HIV-AIDS, HAART

## Abstract

**Background:**

Depression and HIV/AIDS are common mental health problems in people living in refugee camps under stressful conditions. When both conditions occur together in an already vulnerable population living in a deprived situation, they lead to severe health outcomes and complicate patient care. However, there is a shortage of data on the magnitude of depression and related factors among displaced populations living with HIV/AIDS in refugee camps. Therefore, this study aimed to assess the prevalence and factors associated with depression among HIV/AIDS patients living in the Okugu Refugee Camp, Ethiopia.

**Methods:**

A health facility-based cross-sectional study was conducted among a sample of 382 adults living with HIV/AIDS who were on highly active antiretroviral therapy (HAART) at the Okugu Refugee Camp. Data were collected using a structured, interviewer-administered questionnaire and medical chart review based on a standard checklist. The Patient Health Questionnaire-9 (PHQ-9) was used to assess depression. Data were coded and entered into EpiData version 4.6.0.6 and analyzed using SPSS version 23. Descriptive statistics and bivariate and multivariable binary logistic regression analysis were performed. Variables with a p-value less than 0.05 in the final model were considered statistically significantly associated with depression.

**Results:**

A total of 380 study participants took part in the study, with a response rate of 99.4%. More than half of the study participants (58.2%) were female, and the mean age of respondents was 32.71 (SD=7.42). The prevalence of depression among the displaced South Sudanese population living with HIV/AIDS in the Ethiopia Okugu Refugee Camp was 56.8% (95% CI: 51.8%–61.9%). Female sex (AOR = 2.6; 95% CI: 1.24, 6.28), opportunistic infections (AOR = 3.00; 95% CI: 1.75, 7.06), a CD4 count < 200 cells/mm^3^ (AOR = 2.40; 95% CI: 1.78, 8.23), and poor social support (AOR = 4.70; 95% CI: 1.98, 9.79) were significantly associated with depression among the refugees.

**Conclusions:**

The magnitude of depression among refugees living with HIV/AIDS was significantly high. Therefore, regular screening of people living with HIV for depression through integration of mental health services with routine ART services and equipping healthcare providers with essential supplies to deal with the problems of refugees are crucial.

## Introduction

According to the United Nations High Commissioner for Refugees (UNHCR) report, approximately 117.3 million people were forcibly displaced worldwide by the end of 2023, and approximately 75% of them were hosted in low- and middle-income countries, where health systems and other basic services are already overwhelmed (1). These forcibly displaced people are at a higher risk for HIV (2–4), mental health problems (5–7), and social and psychological problems (8, 9).

By the end of 2023, the World Health Organization (WHO) estimated 39.9 million people were living with HIV, and approximately 20.5 million and 1.4 million HIV cases were women and children, respectively. Moreover, the report showed that 26.0 million people living with HIV (PLHIV) in 2023 were from the African region, and the virus has claimed almost 42.3 million lives thus far (10). Studies have shown that the prevalence of HIV is higher among refugees and migrants than in host populations (2, 11, 12). Moreover, migrants experience delayed entry into HIV care and have poorer HIV-related outcomes when compared to native-born populations in their host country due to different barriers (4).

Migrants and refugees are exposed to various stressors that exacerbate pre-existing mental health conditions or contribute to the development of new ones. Depression is the most common mental disorder that affects migrants and refugees (5, 6). It is a mental health problem that presents with a depressed mood, loss of interest or pleasure, decreased energy, feelings of guilt, disturbed sleep or appetite, and poor concentration (13). Globally, it is estimated that 350 million individuals, or approximately 4.4% of the global population, suffer from depression (13, 14). The co-morbidity of depression is nearly twice as prevalent among people living with HIV in Africa as compared to the general population (15). In sub-Saharan countries, 1.57 million people living with HIV/AIDS, or approximately 50% to 60%, are affected by depression (16).

The co-occurrence of depression and HIV/AIDS could result in poor health outcomes due to obstacles to treatment and the deterioration of medical outcomes, including treatment resistance, increased morbidity, and higher mortality rates. Depression among PLHIV is also associated with poor health-related quality of life, poor adherence to antiretroviral therapy (ART), and unsatisfactory viral load suppression (17–19). In addition, depression contributes to loss of productivity and increases both the direct and indirect costs of medical care (20).

Ethiopia has committed to ending the HIV/AIDS epidemic by 2030 (21). To achieve this goal, the health system must adopt innovative strategies that address previously overlooked or neglected challenges (22). Therefore, identifying and treating depression has the potential to improve various HIV-related outcomes, including adherence to ART (23).

Being a forced migrant or refugee and living with HIV is an extremely stressful condition that can hugely contribute to the development or worsening of pre-existing mental health disorders, including depression. However, the burden of depression in migrants or refugees living with HIV and how being a forced migrant and living with HIV contributes to the development or exacerbation of pre-existing mental conditions are not well-documented globally. Furthermore, there is a lack of integrated care and routine mental health screening in refugee camps, leading to missed opportunities for health professionals to identify depression and other mental health problems among migrants or refugees living with HIV/AIDS. Therefore, this study aimed to assess the magnitude of depression and its associated factors among refugees living with HIV/AIDS in the Okugu Refugee Camp, Ethiopia.

## Methods

### Study setting and study design

A facility-based cross-sectional study design was conducted from 20 April to 30 May 2023, among the population displaced from South Sudan due to civil war and residing in the Okugu Refugee Camp, located in the Dimma District, Gambella Regional State, Western Ethiopia. The camp was established in 2013 following the outbreak of violence between confronting parties, which led to the displacement of a significant number of South Sudanese to neighboring countries, including Ethiopia. The majority of inhabitants of the Okugu Refugee Camp are from Jonglei State in South Sudan. Currently, approximately 13,645 people live in the camp, of whom 64% belong to the Anyuak ethnic group and the remaining 36% to the Murle ethnic group. According to the Okugu Refugee Camp Health Center report, 790 adolescents are living with HIV/AIDS, and receiving ART drugs from the health center. In total, 20 healthcare providers are working in the camp’s health center. The health center provides ART clinic services through a separate clinic specifically for people living with HIV/AIDS.

### Population

The study was done among randomly selected adults aged 18 years or older who were receiving highly active ART (HAART) at the ART clinic of the Okugu Refugee Camp Health Center for HIV/AIDS treatment. Patients who were severely ill during data collection and unable to respond were excluded from the study.

### Sample size and sampling procedure

The sample size was determined using Epi Info version 7.1 with the following parameters: statistical power=80%, confidence level =95%, a ratio of exposed to non-exposed 1:1, and an odds ratio of 1.89, which was obtained from a previous study (24). A 5% contingency was added for non-responses, resulting in a final sample size of 382.

The study participants were selected using a systematic sampling technique. The sample frame was generated using medical registration numbers obtained from the ART registry book of the Okugu Refugee Camp Health Centre, after excluding illegible registrations. The sampling interval was calculated by dividing the total population by the total sample size of 382, resulting in a k^th^ interval of two. The first person was selected using a lottery method, and then patients were interviewed every two intervals until the required sample was achieved. The data collection was done by waiting for the sampled patients at the ART clinic when they came for different services.

### Study variables

The dependent variable of the study was depression, categorized as either having depression or not having depression. The independent variables include sociodemographic characteristics (age, sex, marital status, religion, occupation, education status, and income), clinically related factors [opportunistic infections, WHO clinic stage, viral load, ART regimen, duration of illness, CD4 count, adherence to ART, and body mass index (BMI)], social support, perceived stigma, loss of relatives during the conflict, alcohol drinking, cigarette smoking, and khat chewing.

### Data collection tool and procedure

Data were collected using a structured questionnaire through an interview-administered method and medical chart review using a data extraction checklist. The data collection tools were adapted from various similar studies conducted in different parts of the world and modified to fit the local context (25–27). The questionnaire was translated from English into the local languages, Agnuak and Murle, and then back-translated into English by different individuals to ensure consistency. The questionnaire included sections on sociodemographic characteristics, clinically related factors, psychosocial factors, and behavioral factors. Before the actual data collection, the tool was pre-tested on 5% of the sample size among patients who were not part of the main study, and modifications were made accordingly. The data collection was carried out by trained BSc nurses, and the process was supervised by MPH professionals.

Depression was measured using the Patient Health Questionnaire (PHQ-9). The PHQ-9 is a reliable and validated tool in Ethiopia and has demonstrated strong internal consistency (Cronbach’s alpha of 0.85) (25). It has nine items to which participants respond with four scales: not at all (0), several days (1), more than half the days (2), and nearly every day (3). Finally, the responses were summed, and participants were categorized as having depression if they scored 10 or more, otherwise, they were categorized as not having depression (25).

Social support was measured using the Oslo Social Support Scale (Oslo-3), which consists of three items. Based on the total score, respondents were categorized as having poor social support (score 3–8), moderate social support (score 9–11), or strong social support (score 12–14).

Adherence to HAART was determined based on the patient’s recall of their compliance with the prescribed doses over the days preceding the interview. Adherence was calculated by dividing the number of pills taken by the number of pills prescribed to be taken during that period. Then, based on the WHO guideline for measuring optimal treatment adherence, patients who had taken 95% or more of the prescribed pills were considered adherent, while those who had taken less than 95% were classified as non-adherent (28).

Perceived HIV-related stigma was measured using the HIV-Related Stigma Scale (HSS), which consists of eight items. Respondents who scored equal to or above the mean total score were considered to have perceived stigma, while those who scored below the mean were considered not to have perceived stigma (29).

Patients who had at least one of the following opportunistic infections or AIDS-defining cancers: herpes simplex, Salmonella infection, tuberculosis, candidiasis, toxoplasmosis, cytomegalovirus infection, histoplasmosis, cryptosporidiosis, and Kaposi’s sarcoma, either at baseline or after initiation of HAART, were considered to have opportunistic infections or cancers; otherwise, not (29). Alcohol drinkers were defined as those who consumed alcohol more than twice per week, and khat chewers were defined as participants who chewed khat more than twice per week (30).

### Data processing and analysis

The completeness of the data was checked manually before being entered into EpiData software version 4.0.4.6 and subsequently exported to SPSS version 23 for analysis. Descriptive analysis such as frequencies and percentages were calculated. Bivariate binary logistic regression analysis was performed to assess the association of each independent factor with depression. Candidate variables with p-values less than 0.25 in the bivariate analysis were entered into multivariable binary logistic regressions to identify independent factors associated with depression. A backward likelihood ratio with a 0.1 probability of removal was used to develop the model. The goodness of fit of the final model was assessed using the Hosmer–Lemeshow test, and the result indicated a good model fit (p-value=0.789). Variables with a p-value of less than 0.05 in the final multivariable binary logistic regression model were considered statistically significant predictors of depression. Adjusted odds ratios (AORs) with 95% confidence intervals (CIs) were used to estimate the strength of the association. The results are presented in narrative descriptions, tables, and graphs.

## Results

### Sociodemographic characteristics

A total of 380 participants took part in the study, with a response rate of 99.4%. The mean age of the respondents was 32.71 with a standard deviation (SD) of 7.42. More than half of the respondents (58.2%) were female. Regarding religion, the majority (51.0%) were Protestant Christians followed by Muslims (46.6%) (**Table 1**).

**Table 1:** Sociodemographic characteristics of study participants, 2023.

Variable	Category	Frequency (%) N=380	Percentage (%)
Age (in years)	18-34 35-44 45-54 >54	68 174 104 34	17.9 45.8 27.4 8.9
Sex	Male Female	159 221	41.8 58.2
Marital status	Married Single Others	303 55 22	79.7 14.5 5.8
Religion	Protestant Muslim Others	194 177 9	51.0 46.6 2.4
Ethnicity	Agnuak Murle	251 129	66 34
Educational status	No formal education Had a formal education	152 228	40.0 60.0
Occupation	Housewife Incentive workers Private employee Gold miner Students	147 92 59 77 5	38.7 24.2 15.5 20.3 1.3

### Clinical condition of the patients

The CD4 count of 61.1% of the study participants was below 200 cells/mm^3^, and 36.6% of the participants had experienced opportunistic infections. Furthermore, 25.3% of study participants had a viral load of greater than 1,000 copies/ml. Regarding the WHO clinical stage of HIV/AIDS, 27.19% were in stage III, and 9.69% were in stage IV. Finally, 251 participants (66.1%) adhered to their ART (**Table 2**).

**Table 2:** Clinical condition of patients on HAART at Okugu Refugee Camp Health Center, 2023.

Variable	Category	Frequency (n=380)	Percentage (%)
Ever had an opportunistic infection	Yes No	139 241	36.6 63.4
Type of opportunistic infection	Oral Candidiasis Tuberculosis Cytomegalovirus Other	83 34 19 3	59.71 24.46 13.67 2.16
Duration of HIV diagnosis and enrollment	<1 year >1 year	136 244	35.8 64.2
Viral load	≥1000 cell/copies <1000 cell/copies	96 284	25.3 74.7
CD4 count	<200cell/mm3 >200cell/mm3	232 148	61.1 38.9
Current WHO clinical stage	1^st^ stage 2^nd^ stage 3^rd^ stage 4^th^ stage	301 63 14 2	79.2 16.6 3.7 0.5
Type of ART regimen received currently	1^st^ line regimen 2^nd^ line regimen	366 14	96 4
Adherence to ART medication	Adhered Not adhered	251 129	66.1 33.9
Body mass index	<18.5 18.5-24.9 ≥25	106 225 49	27.9 59.2 12.9

### Social support, perceived stigma, and behavior

According to the standard Oslo-3 item classification, 172 respondents (45.2%) had moderate social support. Nearly half (54.2%) of the participants had not experienced perceived stigma. Regarding substance use, 68.7% of respondents consumed alcohol, 26.6% chewed khat, and 69.7% smoked cigarettes (**Table 3**).

**Table 3:** Social support, perceived stigma, and behavior-related factors among adults on HAART at Okugu Refugee Camp Health Center, 2023.

Variable	Category	Frequency (N=380)	Percentage
Social support	Poor Moderate Strong	134 172 74	35.3 45.2 19.5
Perceived stigma	Yes No	174 206	45.8 54.2
Alcohol drinking	Yes No	261 119	68.7 31.3
How often? (n=261)	Once a week 2–4 times a week >5 and more per week	58 92 111	22.2 35.2 42.6
Chewing khat	Yes No	101 279	26.6 73.4
How often? (n=101)	Once a week 2–4 times a week >5 and more per week	23 43 35	22.8 42.5 34.7
Smoking	Yes No	265 115	69.7 30.3
How often? (n=265)	< one pack per day >two packs per day	163 102	61.5 38.5
Loss of biological relatives	Yes No	96 284	25.3 74.7

### Prevalence of depression

**Figure 1:**
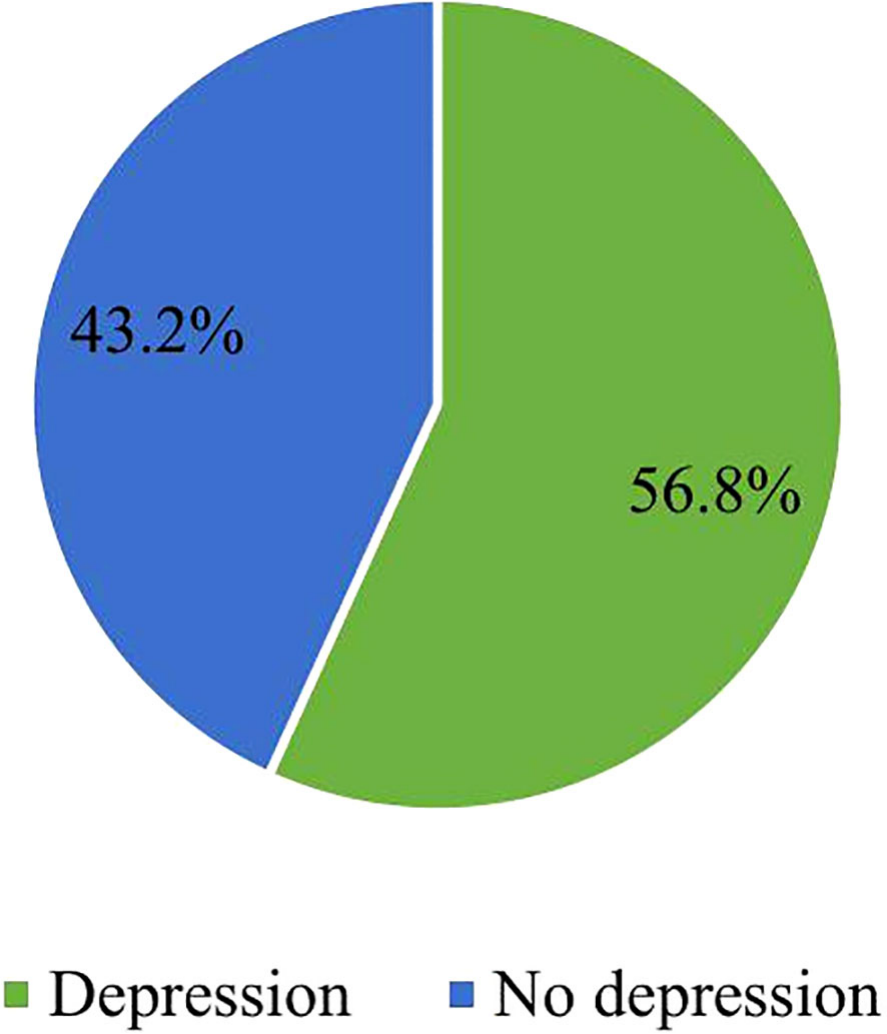
Magnitude of depression among people with HIV-AIDS receiving HAART at Okugu Refugee Camp.

The prevalence of depression among the refugees on HAART at Okugu Refugee Camp Health Center was 56.8% (95% CI: 51.8%–61.9%) (**Figure 1**).

### Factors associated with depression

In the bivariable analysis, variables including sex, presence of opportunistic infections or cancers, current CD4 count, viral load, duration since HIV diagnosis, loss of biological relatives, perceived stigma, social support, smoking, and khat chewing had p-values less than 0.25. These variables were included in the multivariable binary logistic regression to control for potential confounders and identify independent determinants of depression among adult HIV/AIDS patients living in the refugee camp. The multivariate binary logistic regression model revealed that sex, opportunistic infection, CD4 count, and level of social support were statistically significantly associated with depression in patients on HAART at Okugu Refugee Camp (p-value <0.05).

In this study, female patients had 2.6 times higher odds of having depression as compared to male patients (AOR=2.6: 95% CI; 1.24, 6.28). Moreover, patients with HIV/AIDS who had suffered opportunistic infections or cancers were three times more likely to experience depression compared to those who had not suffered opportunistic infections or cancers (AOR=3.0; 95% CI; 1.75, 7.06). In addition, individuals with a CD4 count of less than 200 cells/mm^3^ had 2.4 times higher odds of suffering depression than those with a CD4 count greater than or equal to 200 cells/mm^3^ (AOR=2.4: 95% CI, 1.78, 8.23). The refugees who had poor social support were 4.7 times more likely to be affected by depression as compared to those who had strong social support (AOR=4.7: 95% CI; 1.98, 9.79) (**Table 4**).

**Table 4:** Factors associated with depression among refugees on HAART at Okugu Refugee Camp, 2023.

Variable	Category	Depression		COR	AOR	P value
		Yes	No			
Sex	Male Female	72 (45.3) 153 (69.2)	87 (54.7) 68 (30.8)	1 2.7 (1.29, 6.05)	1 2.6 (1.24, 6.28)	0.002
Opportunistic infections/cancers	Yes No	102 (73.4) 113 (46.9)	37 (26.6) 128 (53.1)	3.1 (1.02, 8.46) 1	3.0 (1.75, 7.06) 1	0.001
Duration of illness (HIV)	<1 year >1 year	64 (47.1) 151 (61.9)	72 (52.9) 93 (38.1)	0.5 (0.81,1.93) 1	0.6 (0.39, 3.41) 1	0.072
CD4 count	<200cell/mm3 >200cell/mm3	145 (62.5) 59 (39.9)	87 (37.5) 89 (60.1)	2.5 (1.89, 9.72) 1	2.4 (1.78, 8.23) 1	0.002
Viral load	≥1,000 cells/copies <1,000 cells/copies	69 (46.4) 146 (37.9)	27 (53.6) 138 (62.1)	2.4 (1.04, 7.15) 1	2.2 (0.88, 6.71) 1	0.060
Loss of relatives	Yes No	75 (78.1) 140 (49.3)	21 (21.9) 144 (50.7)	3.7 (2.33, 8.58) 1	3.5 (1.79, 6.47) 1	0.041
Perceived stigma	Yes No	118 (67.8) 97 (47.1)	56 (32.2) 109 (52.9)	2.4 (1.46, 7.39) 1	2.3 (1.35, 6.37) 1	0.008
Social support	Poor Moderate Strong	89 (66.4) 105 (61.0) 21 (28.4)	45 (33.6) 67 (39.0) 53 (71.6)	4.9 (2.12, 10.45) 3.9 (2.10, 7.79) 1	4.7 (1.98, 9.79) 3.9 (1.87, 9.23) 1	0.004 0.054
Smoking	Yes No	162 (61.1) 53 (46.1)	103 (38.9) 62 (53.9)	1.8 (1.02, 10.67) 1	1.8 (0.92, 8.33) 1	0.093
Khat chewing	Yes No	76 139	25 140	3.1 (1.77, 12.09) 1	3.0 (2.10, 9.87) 1	0.003

## Discussion

This study aimed to assess the magnitude of depression and its associated factors among people living with HIV/AIDS on HAART in Okugu Refugee Camp. The study showed that sex, opportunistic infections or AIDS-defining cancers, CD4 count, and social support were significantly associated with depression. The prevalence of depression was 56.8% (95% CI: 51.8%–61.9%).

This finding is consistent with similar studies conducted in India (31) and North Central Nigeria (32) with 58.75% and 56.7%, respectively. However, it is higher than the prevalence reported in rural South Africa (42.4%) (33) and Malawi (18.9%) (34), and studies carried out in three areas of Ethiopia: Bale (44.9%), Hawassa (48.6%), and Harar (45.8%) (26, 29, 35). The higher prevalence observed in this study could be because the current study was conducted among a refugee population that may have suffered numerous stressors that cause or exacerbate mental health conditions, including depression. The refugees and migrants living with HIV are susceptible to a number of traumatic events including the loss of loved ones, lack of social support, stigma from the host community, rape and other sexual assaults, and limited access to mental health services at camps. Moreover, variations in measurement and sample size may also have accounted for the observed differences.

This study showed that female patients living with HIV were more likely to experience depression compared to their male counterparts. The finding is congruent with other studies conducted in South Africa (36); Khartoum, Sudan (37); and Debre Birhan, Ethiopia (38). Related to sex, there are unique biological factors, hormonal variation, and exposure to several psychosocial burdens that could make women more likely to suffer worry, stress, and depression (39). Moreover, women in refugee settings tend to be exposed to sexual harassment and violence in the household and in the broader community, which can further exacerbate their mental health challenges (40).

The odds of depression were significantly higher in individuals with a CD4 cell count below 200 cells/mm^3^ compared to those with 200 cells/mm^3^ or more. This finding is consistent with studies conducted in Malawi (34) and Ethiopia (38). One possible explanation is that depression can negatively impact the immune system by reducing immunoglobulin, lymphocyte activity, and natural killer cell function (41). Additionally, depression among patients with HIV is often associated with poor adherence to ART medication, which may lead to unsatisfactory viral load suppression and inadequate CD4 cell recovery (17–19).

We found that recent opportunistic infections or AIDS-defining cancers were associated with an increased occurrence of depression. This finding aligns with previous studies conducted in Addis Ababa and South Wollo, Ethiopia (22, 42). A possible explanation is that opportunistic infections worsen the quality of life among people living with HIV, which can consequently lead to poor mental health outcomes. These infections are often linked to hospitalization and diminished functional capacity, which may impact a patient’s psychosocial wellbeing.

This study indicates that individuals who had poor social support had a higher risk of depression. This finding is supported by a study conducted in the Mecha district, Northwest Ethiopia (43). Social interaction among family members and their ability to respond constructively to life transitions play a crucial role in reassuring individuals and maintaining social cohesion (44). People without families to disclose their problems to are unable to receive care and may have increased depressive symptoms (45). Moreover, when social connectedness is lost, the individual may feel isolated and experience heightened feelings of depression.

## Conclusion

This study suggests that the prevalence of depression among refugees living with HIV in the Okugu Refugee Camp is high. Being female and having opportunistic infections or AIDS-defining cancers, a CD4 count of less than 200 cells/mm^3^, and poor social support were identified as significant predictors of depression among people living with HIV attending the ART clinic in the camp. To mitigate the effect of this prevalent mental health condition, healthcare providers should be trained to routinely screen for depression in people living with HIV, with particular attention paid to the identified risk factors. Additionally, integrating mental health services into existing HAART programs should be considered in refugee camps. Moreover, special emphasis should be given to strengthening social support systems for forcibly displaced individuals residing in camps.

## Data Availability

The raw data supporting the conclusions of this article will be made available by the authors, without undue reservation.
